# Supplementary material for: A fault-intrusion-tolerant system and deadline-aware algorithm for scheduling scientific workflow in the cloud
Source: PeerJ Comput Sci. 2021 Nov 2;7:e747. doi: 10.7717/peerj-cs.747 (PMC8576554; doi:10.7717/peerj-cs.747)
Supplement: Supplemental Information 6 [file peerj-cs-07-747-s006.zip › Myproject1.1/docs/org/workflowsim/utils/ClusteringParameters.ClusteringMethod.html]

ClusteringParameters.ClusteringMethod


JavaScript is disabled on your browser.


- Overview
- Package
- Class
- Use
- Tree
- Deprecated
- Index
- Help

- Prev Class
- Next Class

- Frames
- No Frames

- All Classes

- Summary:
- Nested |
- Enum Constants |
- Field |
- Method

- Detail:
- Enum Constants |
- Field |
- Method


org.workflowsim.utils

## Enum ClusteringParameters.ClusteringMethod

- java.lang.Object
- - java.lang.Enum<ClusteringParameters.ClusteringMethod>
  - - org.workflowsim.utils.ClusteringParameters.ClusteringMethod

- All Implemented Interfaces:
  :   java.io.Serializable, java.lang.Comparable<ClusteringParameters.ClusteringMethod>

  Enclosing class:
  :   ClusteringParameters

  ---

    

  ```
  public static enum ClusteringParameters.ClusteringMethod
  extends java.lang.Enum<ClusteringParameters.ClusteringMethod>
  ```

  Supported Clustering Method, by default it is none

- - ### Enum Constant Summary

    Enum Constants

    Enum Constant and Description

    `BALANCED`

    `BLOCK`

    `HORIZONTAL`

    `NONE`

    `VERTICAL`
  - ### Method Summary

    Methods

    | Modifier and Type | Method and Description |
    | --- | --- |
    | `static ClusteringParameters.ClusteringMethod` | `valueOf(java.lang.String name)` Returns the enum constant of this type with the specified name. |
    | `static ClusteringParameters.ClusteringMethod[]` | `values()` Returns an array containing the constants of this enum type, in the order they are declared. |

    - ### Methods inherited from class java.lang.Enum

      `clone, compareTo, equals, finalize, getDeclaringClass, hashCode, name, ordinal, toString, valueOf`
    - ### Methods inherited from class java.lang.Object

      `getClass, notify, notifyAll, wait, wait, wait`

- - ### Enum Constant Detail


    - #### HORIZONTAL

      ```
      public static final ClusteringParameters.ClusteringMethod HORIZONTAL
      ```


    - #### VERTICAL

      ```
      public static final ClusteringParameters.ClusteringMethod VERTICAL
      ```


    - #### NONE

      ```
      public static final ClusteringParameters.ClusteringMethod NONE
      ```


    - #### BLOCK

      ```
      public static final ClusteringParameters.ClusteringMethod BLOCK
      ```


    - #### BALANCED

      ```
      public static final ClusteringParameters.ClusteringMethod BALANCED
      ```
  - ### Method Detail


    - #### values

      ```
      public static ClusteringParameters.ClusteringMethod[] values()
      ```

      Returns an array containing the constants of this enum type, in
      the order they are declared. This method may be used to iterate
      over the constants as follows:

      ```
      for (ClusteringParameters.ClusteringMethod c : ClusteringParameters.ClusteringMethod.values())
          System.out.println(c);
      ```

      Returns:
      :   an array containing the constants of this enum type, in
          the order they are declared


    - #### valueOf

      ```
      public static ClusteringParameters.ClusteringMethod valueOf(java.lang.String name)
      ```

      Returns the enum constant of this type with the specified name.
      The string must match *exactly* an identifier used to declare an
      enum constant in this type. (Extraneous whitespace characters are
      not permitted.)

      Parameters:
      :   `name` - the name of the enum constant to be returned.

      Returns:
      :   the enum constant with the specified name

      Throws:
      :   `java.lang.IllegalArgumentException` - if this enum type has no constant
          with the specified name
      :   `java.lang.NullPointerException` - if the argument is null


- Overview
- Package
- Class
- Use
- Tree
- Deprecated
- Index
- Help

- Prev Class
- Next Class

- Frames
- No Frames

- All Classes

- Summary:
- Nested |
- Enum Constants |
- Field |
- Method

- Detail:
- Enum Constants |
- Field |
- Method
